# Cup positioning and its effect on polyethylene wear of vitamin E- and non-vitamin E-supplemented liners in total hip arthroplasty: radiographic outcome at 5-year follow-up

**DOI:** 10.1007/s00402-022-04424-2

**Published:** 2022-04-10

**Authors:** Josef Baghdadi, Shareef Alkhateeb, Alexander Roth, VITAS-Group, M Jäger, A Busch, S Alkhateeb, S Landgraeber, S Serong, M Haversath, A vonWasen, H Windhagen, T Flörkemeier, S Budde, J Kubilay, Y Noll, KS Delank, J Baghdadi, R Willburger, M Dücker, A Wilke, F Hütter, Marcus Jäger

**Affiliations:** 1 Department of Orthopedics, Trauma, and Reconstructive Surgery, University Hospital Halle (Saale), Halle (Saale), Germany; 2 Department of Orthopedics, Trauma and Reconstructive Surgery, Marienhospital Mülheim an Der Ruhr, Chair of Orthopedics and Trauma Surgery, University of Duisburg–Essen, Essen, Germany; 3 Aesculap AG, Tuttlingen, Germany

**Keywords:** Total hip arthroplasty, CAD-based wear analysis, UHMWPE-XE, Cup placement

## Abstract

**Background:**

Aseptic loosening remains a challenging problem after total hip arthroplasty. Accurate cup placement and supplementation of antioxidants in acetabular liners might reduce material failure rates. The aim of this study is to assess the effect of the cup position on the wear behaviour of UHMWPE-XE and UHMWPE-X liners in vivo using virtual radiographs.

**Methods:**

We conducted a prospective, randomized, controlled, multicenter trial. Clinical data of 372 probands were analyzed. Anteroposterior pelvic X-rays of 324 patients immediately postoperatively and after 1 and 5 years were evaluated by the RayMatch® analysis software regarding cup position and wear behaviour.

**Results:**

Mean cup anteversion was 20.3° (± 7.4) and inclination was 41.9° (± 7.0) postoperatively. 62.3% of all patients had an anteversion and inclination within the Lewinnek safe zone. Anterior and anterolateral approaches led to significantly higher cup anteversion compared to lateral approaches (27.3° ± 5.5; 20.9° ± 7.2; 17.5° ± 6.6; *p* < 0.001 and *p* = 0.001, respectively). Mean anteversion increased to 24.6° (± 8.0) after 1 year (*p* < 0.001). Only one revision occurred because of implant dislocation. Wear rates from UHMWPE-X and UHMWPE-XE did not differ significantly. Anteversion angles ≥ 25° correlated to increased polyethylene wear (23.7 µm/year ± 12.8 vs. 31.1 µm/year ± 22.8, *p* = 0.012) and this was amplified when inclination angles were ≥ 50° (23.6 µm/year ± 12.8 vs. 38.0 µm/year ± 22.7, *p* = 0.062).

**Conclusion:**

Anterior approaches lead to the highest inaccuracy of cup placement, but cup positioning outside the Lewinnek safe zone does not necessarily cause higher dislocation rates. Moreover, mean anteversion increased by approximately four degrees within the first year after operation, which is expected to be functional due to a regularization of pelvic tilt after intervention. Mid-term wear rates of UHMWPE-X and UHMWPE-XE liners are comparable, but steep cup positions lead to significantly increased polyethylene wear. In summary, a re-evaluation of target zones for intraoperative cup positioning might be considered. In the long-term reduced oxidative embrittlement could lead to superior wear behaviour of vitamin E-blended liners.

## Introduction

Total hip arthroplasty (THA) is known to provide great clinical and functional benefits for patients with disabling hip diseases [1]. Most short-term problems center around insufficient stability with subsequent dislocation of the implant. Up to 50% of dislocation take place within the first 3 months [2, 3]. It is among the leading causes for revisions after primary THA [4]. Overall, dislocation rates were found to be between 1 and 5% [5, 6]. To forego this issue several attempts at defining an optimal cup position have been made. Most notable, Lewinnek et al defined a “safe zone” in 1978 [7]. According to this publication, cup anteversion ought to be within the range of 15° ± 10° with cup inclination at 40° ± 10° to minimize dislocations. Up to this day the Lewinnek safe zone is widely in use but also highly disputed [8]. It was shown that several other patient factors are influencing dislocation rates and increased wear as well, including body mass index (BMI), age, gender, surgical approach and used prosthetic components [9–11]. Additionally, it was found that inappropriate cup positioning might cause impingement [12], increased component wear and edge loading [13, 14] as well as liner fractures, leading to osteolysis and aseptic loosening [15, 16].

Aseptic loosening itself is mainly caused by particulate debris due to increased wear of ultra-high molecular weight polyethylene (UHMWPE) acetabular liners [17, 18]. It is the indication for over 50% of all revision operations [19]. In the last decades efforts focused on altering component designs to reduce backside wear and enhancing wear resistance in general by radiation cross-linking of ultra-high molecular weight polyethylene (UHMWPE-X) [20–24] as well as reducing progressive oxidation by adding antioxidants like alpha-tocopherol (vitamin E) (UHMWPE-XE) [25]. Vitamin E concentrations between 0.1 and 1.0 wt% in the unirradiated liner parts seem to extend the oxidative stability of UHMWPE-XE blends [26]. However, in vitro studies also showed that concentrations of more than 0.3 wt% (mass fraction) in the irradiated surface increase wear by impairing cross-linking [27–30]. Vitamin E can either be blended with UHMWPE resin powder prior to consolidation or diffused into UHMWPE after radiation cross-linking. The first manufacturing process results in a homogenous distribution of vitamin E in the polyethylene liner, but may impair in vivo wear behaviour and long-term implant survival [31–33]. The second approach bypasses this problem, but leads to an inhomogeneous vitamin E distribution in the liner and requires subsequent thermal treatment [27, 34, 35]. The benefits of UHMWPE-XE in vivo are yet to be proven, although current studies suggest that vitamin E-blended liners are, in the medium term, at least as reliable regarding revision rate, aseptic loosening and liner fractures as standard UHMWPE in primary total hip and knee arthroplasty [36, 37].

Several methods have been developed to evaluate implant wear and component migration by comparing two conventional X-ray images. Radiostereometric analysis (RSA) is still the gold standard to detect relative micro-movement of prosthetic components in relation to each other and to the bone [38]. The traditional RSA method is very expensive and requires the implantation of tantalum beads with diameters between 0.1 and 1 mm into the bone during operation. Without this referencing material, measurements become difficult to perform and inaccurate. Otherwise, there are several well-established software-based techniques, mainly semi-automated, graphical methods like Martell’s Hip Analysis Suite (HAS), Einzel-Bild-Roentgen-Analyse (EBRA), Poly-Ware and Rontgen Monogrammetric Analysis (ROMAN). As a general rule, they require the placement of reference points in the X-ray image, which results in low reliability [39]. Moreover, complex component shapes like the acetabular cup are approximated as circles. In this study a new, innovative approach, based on virtual computer-aided design (CAD)-based radiographs was used. Beforehand, this method was validated for analysis of polyethylene wear in vivo [40, 41].

## Materials and methods

The VITAS (Vitelene® against Standard UHMWPE-X) study is a prospective, randomized, multicenter trial established in 2011 (PI senior author). Its aim is to assess whether UHMWPE-XE (Vitelene®, sponsor: Aesculap AG, Tuttlingen, Germany) is superior to UHMWPE-X in primary cementless THA regarding in vivo oxidation. Secondary endpoints include clinical and radiological results as mentioned below. In total 400 patients of both genders with advanced hip osteoarthritis and indication for cementless THA were recruited by six different study centers in Germany between 2011 and 2015. A follow-up period of 15 years is planned with evaluation after 5, 10 and 15 years. Exclusion criteria were significant narcosis risk (ASA IV), tumors, drug or alcohol addiction, immunosuppressive therapy, clinically relevant infections, fractures, previous surgeries at the affected hip (osteosynthesis, osteotomy, THA), poor bone quality and relevant deformities (leg length differences > 30 mm, offset reductions > 30 mm). Informed consent, according to the Declaration of Helsinki, was obtained from every patient in written form prior to surgery. The local ethics committee (#11-4845-BO) approved the study and it was registered on Clinicaltrials.gov (#NCT01713062).

### Implants and intervention

Vitelene® is a vitamin E-supplemented highly cross-linked polyethylene liner. It is produced from 0.1% vitamin E and polyethylene polymers by compression molding. Patients were randomized to obtain either a Vitelene® liner or a standard UHMWPE-X (remelted, cross-linking by γ-irradiation [75 kGy], sterilization: ethylene oxide) liner. A cementless hemispheric cup (Plasmacup DC® (Aesculap)) that contains a microporous titanium coating (Plasmapore®) was used. The orthopaedic surgeon was free to define the surgical approach and chose one of five different (standard or short stem) stem types (Metha®, Bicontact®, Trendhip®, Excia®, TRJ®, all Aesculap). Biolox® delta heads (Ceramtec, Plochingen, Germany) with 32 or 36 mm diameter were to be used. All interventions were performed without computer navigation. Intraoperative and postoperative treatment was left to the study centers, but all patients were mobilized 1 day after surgery and participated in physiotherapy. The permitted load varied from 20 kg weight bearing for 6 weeks to immediate full weight bearing.

### Radiographic analysis

**Fig. 1:**
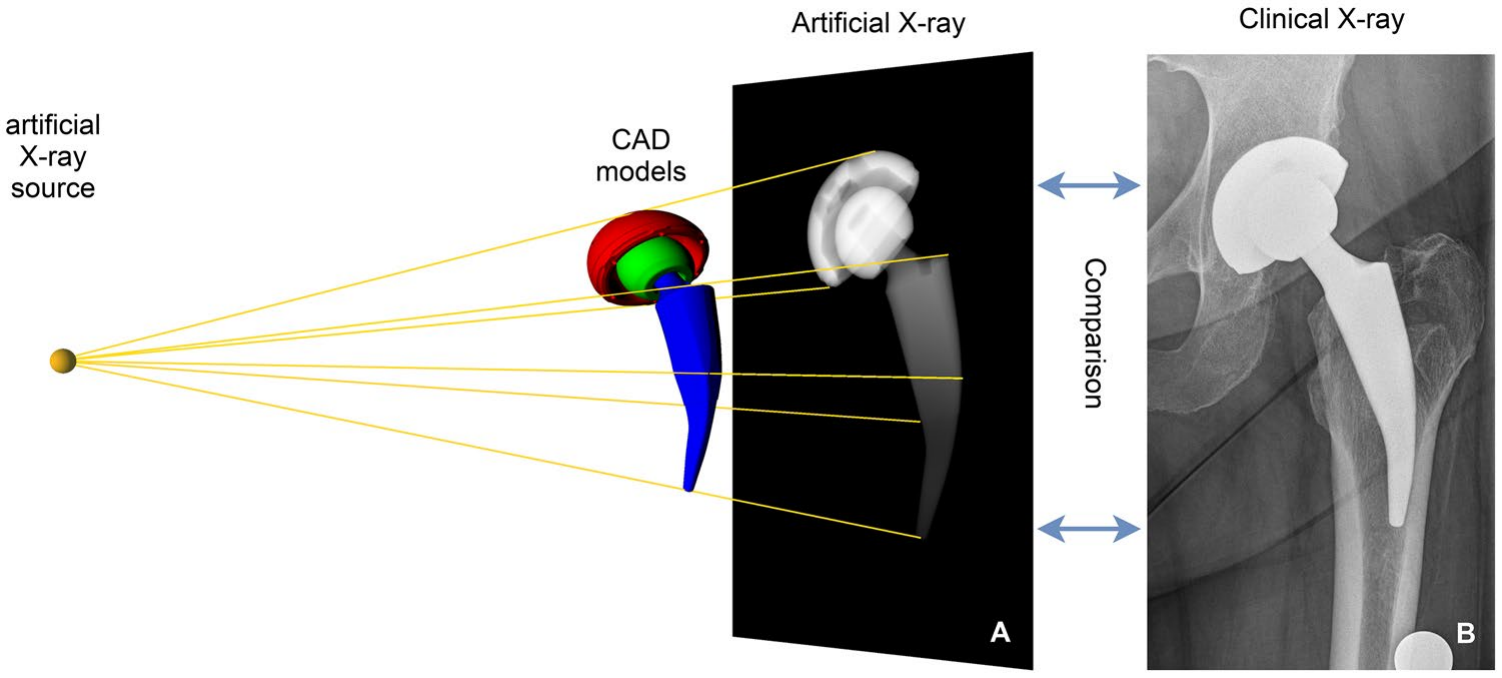
Generation of artificial CAD-based radiographs and comparison to the corresponding clinical X-ray image using the RayMatch® technique (Raylytic, Leipzig, Germany)

For THA parameter measurement the observer-independent analysis software RayMatch® (Raylytic, Leipzig, Germany) was used. It uses CAD data, provided by the manufacturer of the used prostheses components (Aesculap AG, Tuttlingen, Germany) to automatically determine the position and orientation of the prostheses components in anteroposterior pelvic radiographs. Thereby it is possible to measure femoral head penetration (thus polyethylene wear) and cup position (anteversion and inclination). To achieve this, artificial X-ray images of the implants have to be generated by computer simulation. In a first step, the position of the implant in the actual X-ray is automatically identified using a trained neural network. The simulation model mirrors the X-ray set up and consists of an X-ray source, a projection surface (detector) and the prostheses components. Depending on the prosthetic material corresponding attenuation coefficients are assigned to these implants. Vectors reaching from the X-ray source to the detector are calculated. The resulting radiation intensity along these vectors is calculated using the Lambert–Beer law. The intensity at every pixel of the detector corresponds to a value on a normalized grey scale. This creates a realistic virtual (digitally reconstructed) radiograph, which can be compared to a real X-ray image after converting both to so called gradient images that show the alteration of grey values between neighboring pixels in horizontal and vertical direction. The concordance between the two gradient images is determined by normalized cross correlation and the position and rotation of the prostheses components is iteratively optimized in all axes until the similarity of both images reaches a maximum (Fig. 1). This process is called 2D–3D registration. Prior validation showed that RayMatch® is producing results equal to RSA without the need for insertion of tantalum beads [39, 40].

Cup anteversion was defined as the angle between the acetabular axis (centred axis perpendicular to the cup’s opening plane) and the frontal plane in accordance to the radiographic definition of Murray [42]. Accordingly, cup inclination was defined as the angle between the projected acetabular axis on the frontal plane and a vertical reference line. Hip implant wear was quantified as the femoral head penetration (change in relative position between femoral head and acetabular cup between two consecutive time points) in micrometers. The wear rate is the average hip implant wear per year.

### Statistical analysis

A linear mixed model was used to determine the dependency of cup anteversion and cup inclination by time, age, BMI and sex. This included a random intercept per subject and center to compensate for center effects. The time variable consisted of three time points: immediately postoperatively, 12 months and 60 months after surgery. A pairwise post hoc test was applied when a significant time effect was detected and the corresponding *p* values were subsequently corrected by the Tukey method.

The same model as described above was used to model the influence of the surgical approach on cup anteversion and inclination (replacing the time variable with the surgical approach variable).

For wear rate analysis again a linear mixed model was utilized, however, because there were no repeated measurements within subjects, only the study center was used as a random effect. Wear rate was modeled as a dependent variable. Cup inclination and anteversion (dichotomized: in/out of safe zone, ≥ 25° anteversion and ≥ 50° inclination), age, BMI and sex were entered into analysis as independent variables. The model that contained the binary variables ≥ 25° anteversion and ≥ 50° inclination showed a clear pattern of variance inhomogeneity represented by different length of error bars. Because of this a variance term was introduced to allow the variance between the four groups to be different.

*p* values < 0.05 were considered significant. For statistical analysis the software R (version 4.1.0) was utilized.

## Results

Of 400 initially included patients, 28 had to be excluded due to withdrawn consent, intraoperative choice of other implants or randomization errors. Of the remaining 372 probands, 171 (46%) were male and 201 (54%) female. The mean age at time of surgery was 62 years (± 8.2). Mean BMI was at 28.9 kg/m^2^ (± 5.4). 192 patients (52%) received UHMWPE-XE inlays and 180 (48%) received UHMWPE-X inlays. The group characteristics did not differ significantly.

In 38 patients, a direct anterior surgical approach was chosen, 212 patients received an anterolateral approach, 118 a lateral approach and 2 patients each a posterolateral and posterior approach. In 172 patients a Metha® short stem was implanted. In 139 cases a Bicontact® straight stem, in 42 cases a Trendhip® straight stem and in 12 cases an Excia® T stem system was used. A trochanter preserving TRJ® stem was used in seven patients.

Revision surgery was performed in ten cases (2,7%). Seven of these occurred in the UHMWPE-X cohort and three in the UHMWPE-XE group. Three revisions were necessary because of postoperative hemorrhage, two due to periprosthetic fractures, two due to stem loosening and one each because of periprosthetic infection, dislocation and aseptic cup loosening. In only two of these ten cases, explanted inlays were sent in for further analysis. Radiographic images of sufficient quality existed for 324 probands.

**Fig. 2:**
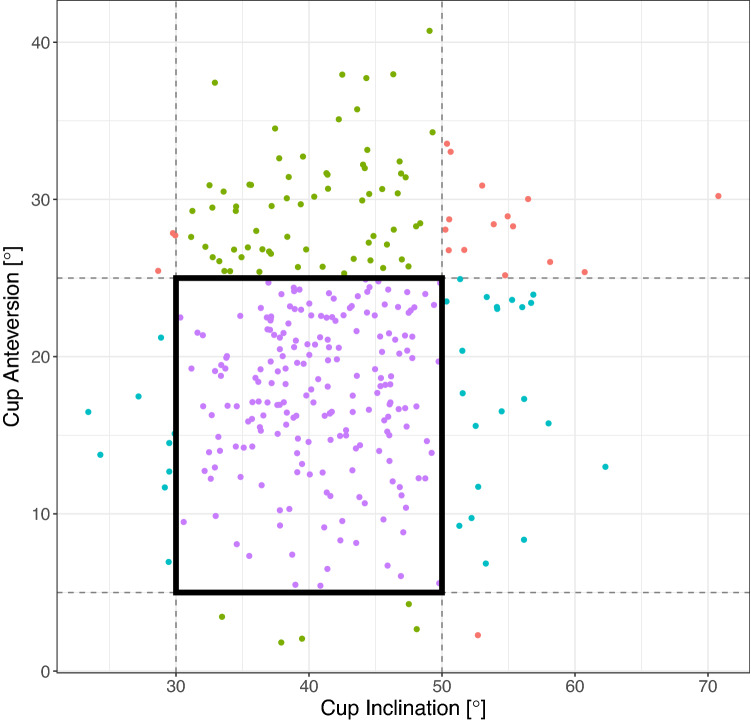
Scattergram of measured postoperative cup anteversion and inclination according to the Lewinnek safe zone (box)

Postoperatively, cup anteversion was measured to be 20.3° (± 7.4) and cup inclination was at 41.9° (± 7.0). A cup anteversion between 5° and 25° was calculated for 232 patients (71.6%) and a cup inclination between 30° and 50° was present in 275 patients (84.9%). In 202 cases (62.3%) anteversion and inclination lay within the Lewinnek safe zone (Fig. 2). In the described single case of dislocation, a cup anteversion of 32.0° and an inclination of 44.2° was measured.

Cup anteversion increased to 24.6° (± 8.0) 12 months after operation (*p* < 0.001). At 5-year follow-up cup anteversion did not change significantly anymore (*p* = 0.999) and was measured at 24.8° (± 8.7). Cup inclination increased to 43.6° (± 7.8) after 12 months (*p* < 0.001). After 5 years, an inclination of 42.1° (± 7.2) was measured, which meant no further significant alteration (*p* = 0.056) (Table 1).

**Table 1:** Changes in measured cup anteversion and inclination over time. *n* = 324

	Post-OP	1 year	5 years	*p* value
Cup anteversion	20.3° (± 7.4)	24.6° (± 8.0)	24.8° (± 8.7)	p_01_ < 0.001 p_05_ < 0.001 p_15_ = 0.999
Cup inclination	41.9° (± 7.0)	43.6° (± 7.8)	42.1° (± 7.2)	p_01_ < 0.001 p_05_ < 0.001 p_15_ = 0.056

*p*_01_—*p* value for change from postoperative to 1 year

*p*_05_—*p* value for change from postoperative to 5 years

*p*_15_—*p* value for change from 1 to 5 years

**Fig. 3:**
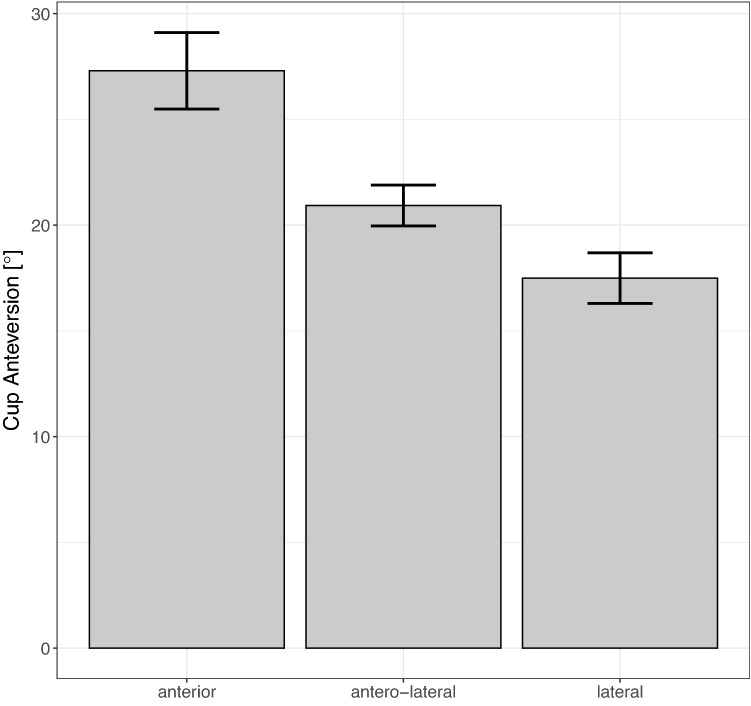
Bar graph of measured postoperative cup anteversion depending on the chosen surgical approach

The chosen surgical approach had a significant effect on postoperative cup anteversion. Especially the anterior approach (27.3° ± 5.5), but also the anterolateral approach (20.9° ± 7.2) led to significantly higher cup anteversion angles compared to a lateral approach (17.5° ± 6.6) (*p* < 0.001 and *p* = 0.001, respectively). In total, 67.7% (± 7.6) of all anterior surgical approaches led to cup anteversions outside the Lewinnek safe zone, compared to 29.3% (± 3.1) of anterolateral approaches and 14.3% (± 3.2) of lateral approaches (Fig. 3). Cup inclination however, was not significantly affected. In a generalized linear mixed model a significant effect of BMI, sex or age on inclination and anteversion depending on the chosen surgical approach could be ruled out.

**Fig. 4:**
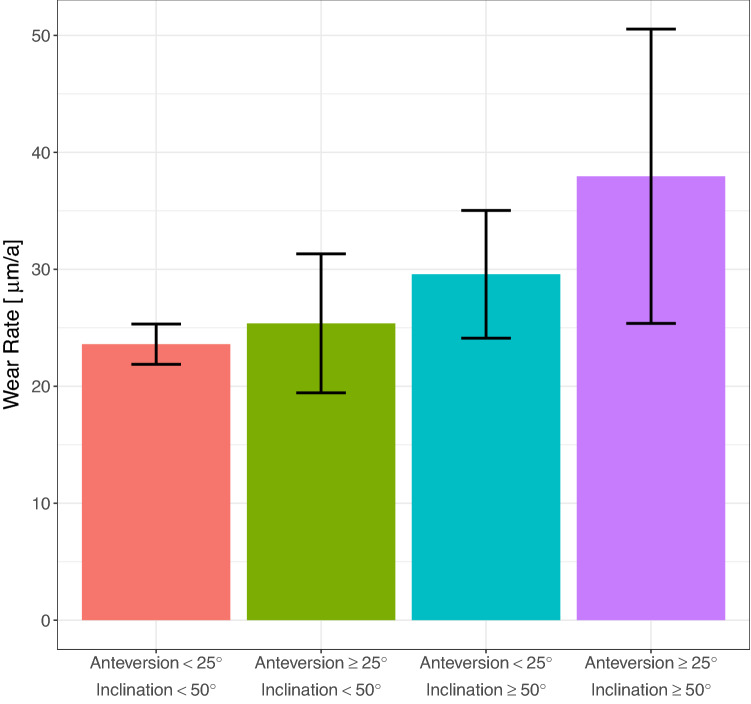
Bar graph of measured annual wear rate within the first 5 years subject to the postoperatively measured cup position

Wear rates from UHMWPE-X (25.4 µm/year ± 15.8) and UHMWPE-XE (26.0 µm/year ± 17.0) did not differ significantly at 5-year follow-up (*p* = 0.748) and were independent of the particular type of implant. In contrast to that, wear rates differed greatly depending on the postoperative cup position. Independent of the liner material, higher anteversion rates, especially more than 25°, correlated to increased polyethylene wear (23.7 µm/year ± 12.8 vs. 31.1 µm/year ± 22.8, *p* = 0.012) and this effect was amplified (although not statistically significant) when inclination was measured to be more than 50° (23.6 µm/year ± 12.8 vs. 38.0 µm/year ± 22.7, *p* = 0.062) (Fig. 4).

## Discussion

Aseptic loosening and dislocation are the leading causes for revisions following THA [4]. In our study only one revision each because of aseptic loosening and dislocation occurred within 5 years.

The Lewinnek safe zone is widely in use as guidance for optimal cup placement and is defined as a cup anteversion within the range of 15° ± 10° with cup inclination at 40° ± 10° [7]. Its meaningfulness is widely disputed since it is based not only on dislocation rates after primary THA, but also after revision cases. In recent years, efforts have been made to redefine implant positioning targets, also taking stem antetorsion and functional aspects like range of motion, into account [43–45].

The single dislocation that was reported had a cup anteversion of 32.0° (outside the “safe zone”) and an inclination of 44.2° (inside the “safe zone”). High cup anteversion, especially combined with high stem antetorsion is known to be a major risk factor for anterior instability [38, 46]. Unfortunately, stem positioning was not determined in our study. It is plausible that increased cup anteversion played a role in the described dislocation event, but it seems likely that it is just one of several contributing factors. In total, only 62.3% of all measured cup positions in our study lay within the Lewinnek safe zone, in 84.9% only inclination was optimal, in 71.6% only anteversion was within the defined range. This is roughly in line with other publications [46, 47], which found cup position to be accurate for inclination in 62–84%, for anteversion 69–79% and for both in 47–58% of examined cases.

In a prior preliminary unicentric publication by our research group, we already established that measured cup anteversion increased within the first year after operation, but changed no further in the following years [36]. We now found the same to be true in our multicenter analysis, where cup anteversion was measured to be 20.3° (± 7.4) and cup inclination was at 41.9° (± 7.0) postoperatively, with an increase to 24.6 (± 8.0) and to 43.6° (± 7.8) after 12 months, respectively. We expect the increase in anteversion to be functional due to a regularization of pelvic tilt as a result of released hip flexion contractures after intervention. It is believed that up to 94% of all patients undergoing THA have some degree of pelvic tilt when positioned supine on the operating table [48]. The amount of postoperative change in pelvic tilt is still unclear, but seems to be less than 5° in general [49–52]. It was found that 1° of pelvic tilt may change measured cup anteversion in anterior–posterior radiographs by as much as 0.7–0.8°, while cup inclination is unaffected [53, 54]. A second radiographic plain was not available, so that pelvic tilt could not be measured in our study and is not accounted for by the used RayMatch® technology. Therefore, the possibility of a cup migration towards flexion must also be considered. Stable osteointegration of titanium cups is known to take place in the early postoperative period [55].

We were able to show that the chosen surgical approach influenced cup anteversion significantly. This correlates to current research, which, similar to our study, shows that the anterior approach leads to higher cup anteversion compared to anterolateral and lateral approaches [13]. It is believed that cup placement through posterolateral and posterior approaches allows for the highest accuracy of cup anteversion. Because each of these two was only used in two patients in our study we cannot validate this. In a multivariate analysis we were able to rule out an influence of age, gender and, most notably, BMI. It appears that the relatively smaller field of view for any given incision size due to the amount of soft tissue does not play a significant role in this effect. In current literature there seems to be no consensus regarding the role of obesity on the accuracy of component placement in THA [13, 56].

Wear rates did not differ significantly between vitamin E-supplemented and non-vitamin E-supplemented inlays at 5-year follow-up and were independent of the particular type of implant used. This matches the findings of other publications [57, 58]. According to Dowd et al wear rates of more than 0.1 mm per year significantly increase the risk of osteolysis [59]. Both groups were considerably below this threshold. In our study revision rates were 3.9% in the UHMWPE-X cohort and 1.6% in the UHMWPE-XE group. Due to the low number of revisions overall and especially the low number of revisions due to aseptic loosening, this difference was not statistically significant. It nonetheless underlines our preliminary findings that UHMWPE-XE liners are not inferior in vivo to standard UHMWPE in the medium-term [36]. This also matches the retrospective analyses of revision rates in total knee arthroplasty by Ftaita et al. [37]. Currently, due to the low number of explanted inlays that reached our research facility, no further conclusions concerning oxidation indices and backside wear can be drawn [24].

We showed that wear rates of UHMWPE-X and UHMWPE-XE liners significantly depend on cup anteversion and that they are further influenced by cup inclination. Several research papers show that cup malpositioning can lead to increased wear in cups with metal-on-polyethylene, metal-on-metal and ceramic-on-ceramic bearings [14, 60, 61]. This seems especially important in hard-on-hard bearings, where wear rates were found to be 10 to 30 times as high when cup abduction exceeded 55°. This may be explained by eccentric sliding of the femoral head, the weight bearing taking place on a smaller contact patch that approaches the edge of the liner and a reduction of fluid film lubrication with steeper cup position. Some studies with less sensitive radiographic measurements might fail to detect this effect in vivo, due to the overall smaller amounts of wear observed in current UHMWPE-X liners [62]. The influence of femoral and combined anteversion on polyethylene wear was shown in a recent study [63]. To our knowledge, the effect of increased cup anteversion on wear behaviour has not been shown yet. It seems possible that a steep cup position, not only in the frontal plain but in general, might affect wear behaviour due to the reasons mentioned above.

Possible impacts on polyethylene liner wear that were not examined by us include femoral head size, acetabular liner offset and individual patient activity level. The in vivo effects of the mentioned parameters are still unclear. While linear wear rates of UHMWPE-X liners seem not to be influenced by larger femoral head size, volumetric wear might surge [64]. Research on the in vivo impact of acetabular liner offset on wear behaviour is rare, nevertheless one study showed that higher offsets might lead to increased volumetric and linear wear [65]. It is also conceivable that higher physical activity, with more gait cycles occurring, might lead to increased liner wear [66].

The design of our study as a prospective, randomized multicenter trial is a major strength. Limiting factors include the unknown pelvic parameters as well as the unknown position of the femoral stem. As mentioned, a second radiographic plain was not available in our study so that especially measurements of the cup anteversion could be adversely affected [67]. Moreover, femoral head size, offset and activity level might affect acetabular linear wear, but were not analyzed in this trial. Of the 400 initially included probands, 28 had to be excluded due to withdrawn consent, intraoperative choice of other implants or randomization errors. Moreover, radiographic images of sufficient quality existed for only 324 probands. The use of the RayMatch® technique for reliable, observer-independent virtual CAD-based analyzation of X-ray images ensured highly accurate measurements of radiographic parameters, but may impede comparability to other studies.

## Conclusion

Wear rates of UHMWPE-X and UHMWPE-XE liners in THA did not differ significantly at 5-year follow-up. In the long term, we assume that reduced oxidative embrittlement could lead to superior wear behaviour. Across both groups high cup anteversion angles (≥ 25°) led to significantly increased polyethylene wear. High cup inclination (≥ 50°) influenced wear behaviour further, but its effect was not statistically significant at 5-year follow-up. We expect this effect to become more apparent over time.

Acetabular cup positioning regarding anteversion was significantly dependent on the chosen surgical approach, with anterior approaches leading to the highest inaccuracy. The reason for this remains unclear, since obesity could not be related to a higher risk of cup malpositioning.

Although only 62.3% of all patients had acetabular cups placed within the Lewinnek safe zone, only one revision took place due to implant dislocation. Moreover, measured cup anteversion increased by approximately four degrees within the first year after operation and is expected to be functional due to a regularization of pelvic tilt after intervention. A re-evaluation of target zones for intraoperative cup positioning might, therefore, be considered.

## Data Availability

All patient-related data were collected by file research from the archives of the participating centers.

## Declarations

### Patient confidentiality

Data were protected according to the U.S. Health Insurance Portability and Accountability Act (HIPAA).
